# Alpha-methylacyl-coenzyme A racemase expression in neuroendocrine neoplasms of the stomach

**DOI:** 10.1007/s00428-012-1272-5

**Published:** 2012-07-11

**Authors:** Alexey Annenkov, Ken Nishikura, Koji Domori, Yoichi Ajioka

**Affiliations:** 1 Division of Molecular and Diagnostic Pathology, Graduate School of Medical and Dental Sciences, Niigata University, Niigata, Japan; 2 Division of Molecular and Functional Pathology, Graduate School of Medical and Dental Sciences, Niigata University, 1-757 Asahimachi-dori, Chuo-ku, Niigata, 951-8510 Japan

**Keywords:** Alpha-methylacyl-coenzyme A racemase, Stomach, Neuroendocrine tumour, Neuroendocrine carcinoma

## Abstract

The enzyme alpha-methylacyl-coenzyme A racemase plays an important role in the beta-oxidation of branched-chain fatty acid and its derivatives. It has been used to detect prostatic adenocarcinoma and high-grade intraepithelial neoplasia, and recently also as a marker for other neoplasms, including those of the genitourinary system, breast, upper and lower gastrointestinal tract and their precursor lesions. We assessed expression of alpha-methylacyl-coenzyme A racemase by immunohistochemistry in neuroendocrine tumours of the stomach to determine differences in the incidence and pattern of expression among different types of gastric neuroendocrine tumours. While none of the grade 1 neuroendocrine tumours were immunoreactive, 67 % of grade 2 neuroendocrine tumours and 90 % of neuroendocrine carcinomas were positive for alpha-methylacyl-coenzyme A racemase. Furthermore, an adenocarcinoma component was found in 72.5 % (37 of 51) of neuroendocrine carcinomas, whereas none of the grade 1 and 2 neuroendocrine tumours contained an adenocarcinoma component. In 83 % of neuroendocrine carcinomas, the adenocarcinoma component was positive for alpha-methylacyl-coenzyme A racemase, and both adenocarcinoma and neuroendocrine carcinoma components stained positively in 78 % of these cases. Our results indicate that alpha-methylacyl-coenzyme A racemase is a useful marker for distinguishing between grade 1 (negative) and grade 2 neuroendocrine tumours, and neuroendocrine carcinoma of the stomach (frequently positive). Different patterns of alpha-methylacyl-coenzyme A racemase expression between gastric neuroendocrine tumours and neuroendocrine carcinoma suggest that these might develop via different tumourigenic pathways.

## Introduction

Neuroendocrine neoplasms generally show characteristic histological features, such as trabecular, sheet-like and solid alveolar architecture, and neuroendocrine granules in the cytoplasm of tumour cells, which sometimes contain hormonal products. According to the latest World Health Organization (WHO) classification of digestive system tumours (fourth edition, 2010), gastric neuroendocrine neoplasms are divided into neuroendocrine tumour (NET) and carcinoma (NEC), and the former is further divided into grade 1 NET (NET G1) and grade 2 NET (NET G2). This classification is based on the proliferative activity of tumour cells. NET G1, NET G2 and NEC of the stomach correspond to what was formerly called carcinoid tumour (synonymous with well-differentiated endocrine tumour), atypical carcinoid tumour and endocrine cell carcinoma (synonymous with small cell carcinoma), respectively. Gastric NET G1 is usually composed of uniform small cells with little atypia, limited growth within the submucosa and rare angioinvasion or distal metastasis, corresponding clinically with low-grade malignant potential and favourable prognosis. NET G2 is also known as a malignant carcinoid and sometimes demonstrates aggressive biological behaviour [1]. NEC is predominantly composed of atypical cells, larger than those of NET with conspicuous mitotic figures, and demonstrates aggressive biological behaviour with frequent angioinvasion, distant metastasis and poor prognosis. Ki67 and p53 are reported to be helpful markers to distinguish between neuroendocrine cell neoplasms, but the histopathological distinction between NET G1 and NET G2, or between NET G2 and NEC [2], remains difficult.

Alpha-methylacyl-coenzyme A racemase (AMACR) is an enzyme that plays an important role in the beta-oxidation of branched-chain fatty acids and their derivatives [3–5]. AMACR was originally identified in mitochondria and peroxisomes of rat liver cells. Given the involvement of AMACR in the metabolism of lipids, it was speculated that overexpression of this protein might lead to alterations in the balance of cellular oxidants, which in turn might contribute to the pathogenesis of neoplasms. AMACR has been primarily used to detect prostatic adenocarcinoma and high-grade intraepithelial neoplasia [6, 7], and recently also as a differential and prognostic marker of several other neoplasms and their precursor lesions, including those from the prostate, genitourinary system, breast and upper and lower GI tract [7–21].

As yet, only two reports have considered AMACR expression in neuroendocrine neoplasms [17, 22], and only five reports mention AMACR expression in gastric neoplasms [18–21, 23]. These studies found that AMACR expression distinguishes between adenoma and/or ordinary adenocarcinoma and non-neoplastic conditions, and that AMACR expression may depend on tumour differentiation. However, none of these studies mention gastric NET or NEC. The aim of our study was to clarify the differences in the incidence and patterns of immunohistochemically determined expression of AMACR between NET G1, NET G2 and NEC of the stomach.

## Materials and methods

### Case selection

For the present study, 82 cases of gastric neuroendocrine neoplasms, registered between 1985 and 2011 at the Division of Molecular and Functional Pathology, Niigata University Graduate School of Medical and Dental Sciences, Niigata City, Japan, were selected. The tumours came from 1 Russian and 80 Japanese patients, who had undergone endoscopic resection or surgical resection without systemic adjuvant therapy. All samples had been fixed in 10 % formalin solution and embedded in paraffin. Clinical information was obtained from the medical records of each patient.

Endocrine differentiation of tumour cells was confirmed by diffuse and intense immunoreactivity of at least one of well-known endocrine markers, including chromogranin A, synaptophysin and neural cell adhesion molecule (NCAM, CD56).

The neuroendocrine cell neoplasms were divided into three groups according to the 2010 WHO classification [1]: (1) NET G1 (22 cases), with a mitotic count of less than two per ten high-power fields (HPF) and/or a Ki67 index ≤2 %; (2) NET G2 (9 cases), with a mitotic count of 2–20 per ten HPF and/or a Ki67 index of 3–20 %; and (3) NEC (51 cases), with a mitotic count >20 per ten HPF and/or a Ki67 index >20 %. The study was approved by the ethics committee of Niigata University Graduate School of Medical and Dental Sciences.

### Histological diagnosis

The diagnosis of gastric neuroendocrine neoplasms was confirmed by two pathologists (AA and KN), who reviewed the clinical information and histological slides. The histological analysis recorded the following: depth of tumour invasion, lymphatic invasion, angioinvasion, mitotic frequency and lymph node metastasis. Lymphatic invasion and angioinvasion were confirmed via immunostaining for D2-40 and CD34.

### Immunohistochemistry

The most invasive area of each tumour was selected, and the corresponding paraffin block was cut into consecutive 3-μm sections for immunostaining, after a representative haematoxylin and eosin (H&E) section was stained.

Immunohistochemical analysis was performed via the high polymer method, using the Histofine MAX-PO (MULTI) kit (Nichirei Biosciences Inc., Tokyo, Japan). Sections were deparaffinized and rehydrated, then microwaved at 98 °C for seven cycles of 3 min (i.e. for synaptophysin, CD34, D2-40, and AMACR) or autoclaved at 121 °C for 20 min (i.e. for Ki-67, chromogranin A, NCAM and p53) in 10 mmol/L of citrate buffer (pH 6.0, for all antibodies except AMACR) or 1 mmol/L Tris–EDTA buffer (pH 9.0, for AMACR) to retrieve antigenic activity. Endogenous peroxidase activity was blocked by 0.3 % hydrogen peroxide in methanol for 20 min. In all sections, except Ki-67, non-specific binding was blocked with 10 % normal serum. Sections were incubated overnight with the following primary antibodies: mouse monoclonal antibodies against chromogranin A (DAK-A3, dilution at 1:500, Dako, Glostrup, Denmark), NCAM (1B6, 1:100, Novocastra, Newcastle, UK), CD34 (QBEnd-10, 1:200, Dako), D2-40 (D2-40, 1:200, Signet Laboratories, Dedham, MA, USA), p53 (PAb1801, 1:40, Novocastra) and Ki67 (MIB-1, 1:200, Dako), as well as rabbit monoclonal antibodies against synaptophysin (1:100, Dako) and AMACR (p504s, clone 13H4, 1:100, Dako). Sections were then incubated with Histofine Simple Stain MAX-PO (MULTI) (Nichirei Biosciences Inc.) for 30 min at room temperature. Antibody–antigen reactivity was visualized with diaminobenzidine, and sections were lightly counterstained with haematoxylin. Finally, the sections were dehydrated with graded ethanol and xylene and mounted with coverslips.

### Interpretation of AMACR immunohistochemical expression

**Fig. 1:**
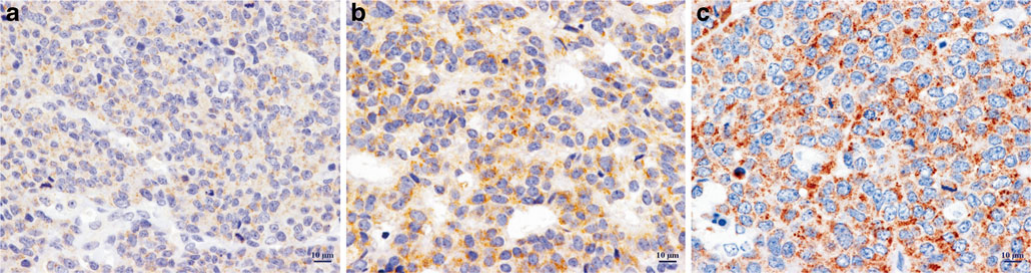
Intensity of AMACR immunohistochemical staining; weak (**a**), intermediate (**b**) and strong (**c**)

Sections from ten cases of prostate cancer served as positive control for AMACR, whereas sections incubated with non-immune mouse IgG served as negative controls. AMACR expression was considered positive when the cytoplasm of >5 % of tumour cells was stained. Positive AMACR staining was divided into three patterns: strong (intense and granular cytoplasmic stain), weak (faint and diffuse cytoplasmic stain) and intermediate (mixture of strong and weak patterns) (Fig. 1).

### Interpretation of Ki67 index and p53 immunohistochemical expression

The Ki67 index was determined via MIB-1 antibody staining and was expressed as a percentage of 1,000 tumour cells counted in areas with strongest nuclear labelling (“hot spots”). Expression of p53 protein was classified as follows: (−) negative, (+) scattered positive cells, (++) focal aggregates of positive cells and (+++) positive cells occurring in most tumour cells. The staining patterns (++) and (+++) were considered as overexpression of p53 protein, consistent with our previous report [2].

### Statistical analysis

Differences between groups were determined via Fisher's exact and *χ* ^2^ tests. All statistical analyses were conducted with PASW statistics 17.0 (SPSS Japan Inc. Tokyo, Japan). A *p* < 0.05 was considered statistically significant.

## Results

### Clinicopathological features

The clinicopathological features of the studied endocrine neoplasms of the stomach are summarized in Table 1. The three types of gastric neuroendocrine neoplasm demonstrated significant differences in tumour size, invasion into the muscular layer or deeper, mitosis, lymphatic invasion, angioinvasion and lymph node metastasis.

**Table 1:** Clinicopathological features of neuroendocrine neoplasms of the stomach

	NET G1 (*n* = 22)	NET G2 (*n* = 9)	NEC (*n* = 51)	*p* value		
				NET G1 vs NET G2	NET G1 vs NEC	NET G2 vs NEC
Sex, male/female	12:10	7:2	40:11	NS	NS	NS
Age (years), average ± standard deviation	57.6 ± 11.7	56.8 ± 15.0	69.6 ± 9.8	NS	0.001	0.01
Tumour size (mm), median (quartile range)	6 (4–10)	20 (8–30)	48.5 (30–61)	NS	<0.001	0.002
mp invasion	0/22 (0 %)	4/9 (44 %)	39/51 (76 %)	0.005	<0.001	NS
Mitosis (per ten HPF), mean (range)	0 (0–1)	2 (1–4)	25 (15–43)	<0.001	<0.001	<0.001
Lymphatic invasion	0/22 (0 %)	6/9 (67 %)	47/51 (92 %)	<0.001	<0.001	NS
Angioinvasion	0/22 (0 %)	4/9 (44 %)	39/51 (76 %)	0.005	<0.001	NS
LN metastasis	0/9 (0 %)	3/5 (60 %)	33/48 (69 %)	NS	<0.001	NS

*NET* neuroendocrine tumour, *NEC* neuroendocrine carcinoma, *NS* not significant, *mp* muscularis propria, *HPF* high-power field, *LN* lymph node

### Histological features

**Fig. 2:**
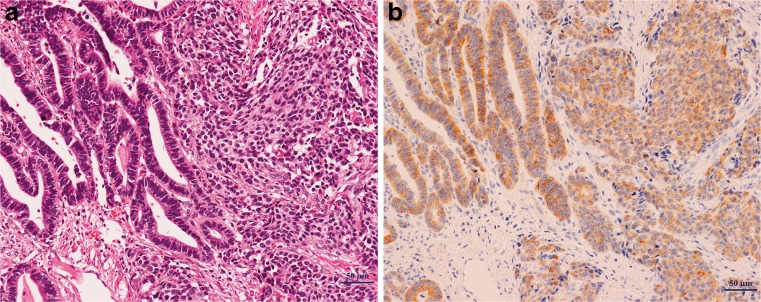
Immunoexpression of AMACR in adenoNEC case. The adenocarcinoma part and NEC part showed histologic continuity (**a**) and similar strong immunoexpression pattern of AMACR (**b**)

An adenocarcinoma component was associated with 37 of 51 (73 %) NEC cases (adenoNEC), and 12 of 37 (32.4 %) cases were diagnosed as mixed adenoneuroendocrine carcinoma (MANEC), a special type of adenoNEC with either component of adenocarcinoma and NEC exceeding 30 % according to the current WHO classification [1]. The remaining 14 cases demonstrated NEC without any signs of adenocarcinoma. Specifically, the adenocarcinoma component that was associated with NEC was limited to the mucosa (68 %, 25 of 37) or submucosa (32 %, 12 of 37), and 34 of 37 cases (92 %) were of well to moderately differentiated adenocarcinoma. Furthermore, 89 % of the differentiated adenocarcinoma components were histologically continuous with NEC components in the submucosal layer (Fig. 2). Conversely, no NET G1 or G2 cases contained an adenocarcinoma component (0 of 22 and 0 of 9).

### AMACR expression in the non-neoplastic epithelium

AMACR was not expressed in the non-neoplastic gastric mucosa surrounding each tumour. Intestinal metaplasia was weakly positive for AMACR staining in <5 % of cells of the 9 % of cases that were considered negative.

### AMACR expression in NET G1, NET G2 and NEC

**Fig. 3:**
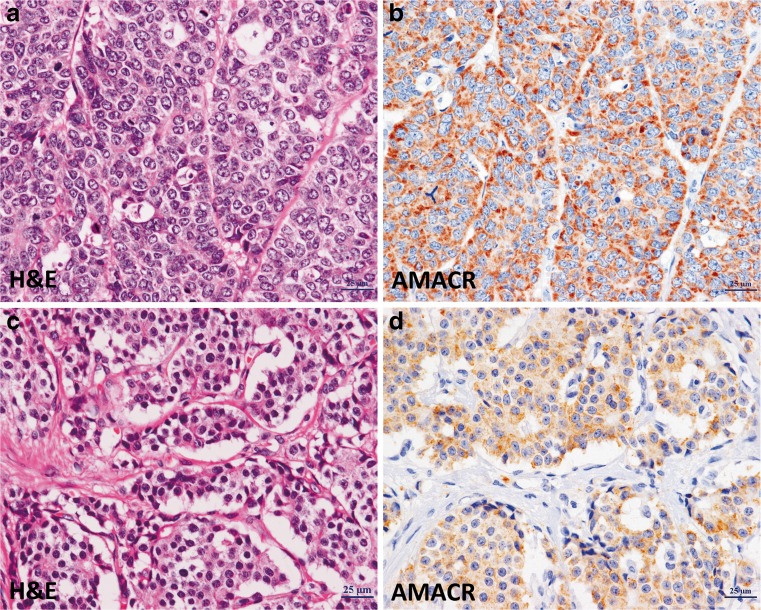
Immunohistochemical staining of NEC and NET G2. NEC (**a**, H&E) demonstrates strong immunoexpression of AMACR (**b**), and NET G2 (**c**, H&E) shows intermediate intensity of AMACR expression (**d**)

AMACR expression levels in gastric neuroendocrine tumours are summarized in Table 2. While none of the NET G1 cases expressed AMACR (0 of 22), 67 % (six of nine) of NET G2 cases expressed AMACR. Specifically, there was strong staining in 33 % (two of six), intermediate staining in 17 % (one of six) and weak staining in 50 % (three of six) of NET G2 (Fig. 3). Furthermore, AMACR was expressed in 90 % (46 of 51) of NEC cases, where 50 % (23 of 46) showed strong staining, 35 % (15 of 46) showed intermediate staining and 15 % (7 of 46) weak staining. Of the NEC cases associated with adenocarcinoma (adenoNEC), the adenocarcinoma component expressed AMACR in 84 % (31 of 37) and the NEC in 89 % (33 of 37). In adenoNEC cases, there was no significant difference in AMACR expression between MANEC and non-MANEC (91.6 vs 80.0 %). Interestingly, 93 % (13 of 14) of NEC without an adenocarcinoma component was positive for AMACR. The adenocarcinoma and NEC components were both positive in 78 % (29 of 37) of cases (Fig. 2). The AMACR expression status was concordant between the adenocarcinoma and NEC component in 83.8 % (31 of 37) of cases, both positive in 78 % (29 of 37) and both negative in 5.5 % (2 of 37) (Fig. 2). For adenoNEC cases, AMACR staining was concordant between the adenocarcinoma and NEC components in 52 % (15 of 29), and the staining patterns were similar (i.e. strong–intermediate, intermediate–weak) in 86 % (25 of 29) of cases. AMACR staining was strong for both adenocarcinoma (15 of 31, 48 %) and NEC (23 of 46, 50 %) (Fig. 2). There were no significant differences between the AMACR-positive and AMACR-negative NEC, and NET G2 cases with respect to clinical features, including tumour size, depth of invasion, lymphatic invasion, angioinvasion and lymph node metastasis (Table 3).

**Table 2:** AMACR expression in neuroendocrine neoplasms of the stomach

Type of tumour	Number	AMACR expression				*p* value
		Weak	Intermediate	Strong	Total	
NET G1	22	0	0	0	0	<0.001
NET G2	9	3 (33.3 %)	1 (11.1 %)	2 (22.2 %)	6 (66.7 %)	
NEC	51	7 (13.7 %)	16 (31.4 %)	23 (45.1 %)	46 (90.2 %)	

*NET* neuroendocrine tumour, *NEC* neuroendocrine carcinoma

**Table 3:** Relationship between the AMACR-positive and AMACR-negative cases of NEC and clinical features

	AMACR positive	AMACR negative
Tumour size (mm), median (quartile range)	49 (18–61)	57 (20–60)
mp invasion	35/46 (76 %)	4/5 (80 %)
Lymphatic invasion	43/46 (93 %)	4/5 (80 %)
Angioinvasion	35/46 (76 %)	4/5 (80 %)
LN metastasis	30/43 (70 %)	3/5 (60 %)

*mp* muscularis propria, *NEC* neuroendocrine carcinoma, *LN* lymph node

### Correlations between Ki67-index, p53 expression and AMACR expression

Expression of p53 and Ki67 index are presented in Table 4. Overexpression of p53 and AMACR expression correlated in neuroendocrine tumours of the stomach. In NET G1 cases, both p53 and AMACR were negative. In NEC cases, p53 overexpression (67 %) and AMACR expression (90 %) often concurred (67 %). However, in NET G2 cases without overexpression of p53, 67 % (six of nine) of cases expressed AMACR. The Ki67 showed a linear correlation with AMACR expression in NET G1, NET G2 and NEC.

**Table 4:** Correlation between Ki67 index, p53 overexpression and AMACR positivity in neuroendocrine neoplasms of the stomach

Type of tumour	Ki67 index, mean (range)	p53 overexpression (*n*)	AMACR positivity (*n*)
NET G1	1.7 % (1.5–2)	0 (0/22)	0 (0/22)
NET G2	7.2 % (4.5–10)	0 (0/9)	66.7 % (6/9)
NEC	67.1 % (55.4–78.9)	66.7 % (34/51)	90.2 % (46/51)

*NET* neuroendocrine tumour, *NEC* neuroendocrine carcinoma

## Discussion

Although AMACR is expressed in numerous tumour types, its expression has never been studied in neuroendocrine neoplasms of the digestive system. There are only two papers showing significant AMACR expression in neuroendocrine neoplasms of the lung. In the present study, the incidence and pattern of immunohistochemical expression of AMACR in NET G1, NET G2 and NEC of the stomach were investigated.

AMACR overexpression has been observed in prostate and colorectal carcinomas, and linked to a high-fat diet [13]. However, the exact mechanism by which a high-fat diet might contribute to tumourigenesis in these organs remains unclear. Furthermore, AMACR expression may not be limited to tumours linked to dietary factors and may be involved in carcinogenesis via a degradation pathway of branched-chain fatty acids, or an epiphenomenon. The pathologenetic link between dietary branched-chain fatty acids and cancer has not yet been determined, and it remains unclear why branched-chain fatty acids appear to be more carcinogenic than straight-chain fatty acids. Some investigators have speculated that the overexpression of AMACR may lead to alteration in the balance of cellular oxidants, resulting in the production of reactive oxygen species, which leads to oxidative stress, and then DNA damage [24].

In the present study, we found that AMACR expression was significantly higher in NEC (90 %) and NET G2 (67 %) than in NET G1 (0 %), and that AMACR expression correlated with the Ki67 index (Table 4). These findings suggest that AMACR might be a good marker for differentiating between these neoplasms. Overexpression of p53 has been considered to be the best marker to distinguish between NEC and NET. Indeed, 67 % of our NEC but no NET cases overexpressed p53. Furthermore, AMACR was more often positive than p53 (90 vs 67 %) in NEC, and furthermore, AMACR was expressed in 67 % of NET G2, suggesting that AMACR may better discriminate between grade 1 and 2 gastric neuroendocrine neoplasms (i.e. not only between NEC and NET, but also between NET G1 and NET G2). Only a few studies have been performed regarding the prognostic significance of AMACR expression in gastric tumours, and it is still controversial [18–20]. We did not find a correlation between aggressive features, such as lymphatic invasion or lymph node metastasis, and AMACR expression in NET G2 and NEC. This may be due to the small number of samples, and future study with a higher number of cases, especially of NET G2, might clarify this issue.

Many gastric NET cases are formed by aggregation of endocrine cell micronests, predominantly composed of enterochromaffin-like cells, and caused by the trophic effect of hypergastrinemia, which occurs upon extensive atrophy of the fundic gland mucosa [25]. Patterns of AMACR expression concurred between adenocarcinoma and NEC components of adenoNEC. The absence of an adenocarcinoma component in NET G1 and G2 cases might suggest not only the histological features, but also the tumourigenic pathway between NET and NEC are different. The possibility that NEC originates from a preceding adenocarcinoma component might be considered.

Shilo et al. reported overexpression of AMACR in pulmonary neuroendocrine tumours, with AMACR expression in 72 % (31 of 43) of typical carcinoids, 52 % (15 of 29) of atypical carcinoids, 70 % (16 of 23) of large cell neuroendocrine carcinomas and 51 % (32 of 63) of small cell carcinomas [22]. Jiang et al. reported that pulmonary small cell carcinomas (zero of five) and pulmonary carcinoid tumours (zero of ten) are negative for AMACR [17]. These data suggest that the malignant potential of pulmonary carcinoids is different from that of gastric carcinoids, as reflected in AMACR staining.

One important limitation of our study is that we used a small number of specimens, especially of NET G2, and therefore, additional studies are necessary to further clarify the overexpression of AMACR in the development of NEC and NET G2, as well as the use of AMACR in differentiating between various neuroendocrine neoplasms of the stomach.
